# WHAT’S NEW IN AGING AND EMOTIONAL EXPERIENCE RESEARCH? NEW QUESTIONS, METHODS, AND SAMPLES

**DOI:** 10.1093/geroni/igae098.1990

**Published:** 2024-12-31

**Authors:** Jennifer Stanley, Eric Allard

**Affiliations:** Chair; Discussant

## Abstract

Older adults tend to report greater control over their emotions and higher well-being, compared to younger adults (Carstensen et al., 2011). Evidence for this phenomenon is robust, but the mechanisms and boundary conditions of improved emotional experience with age are less well understood. This symposium presents four innovative papers that tackle these questions using new methods, asking new questions, and extending to new samples. First, Hamilton and Allard argue for the utility of estimating emotion networks for understanding age differences and similarities in the co-occurrence of emotions. Next, Kravljaca and Stanley investigated age differences in facial feedback effects when posing angry and happy facial expressions. Contrary to expectations based on the Maturational Dualism hypothesis (Mendes, 2010), both young and older adults exhibited increases in angry ratings following the posing of an anger expression. Then, Amanambu and Allard investigated whether specific facets of mindfulness relate to age-related decreases in negative affect in a lifespan sample in Nigeria. They found that the Describing facet mediated the relationship between age and negative affect. Overall, they report similar findings in the Nigerian sample to what has been shown in Western samples. Finally, Behrend and colleagues report an online study that explored whether young and older adults consider specific emotions useful for accomplishing social goals. Both age groups endorsed the most useful emotion for each type of goal, suggesting that there is age similarity in the knowledge of utilitarian emotions in the social domain. These papers present new frontiers in emotions and aging research.

